# Oxygen-Controlled Dynamic Evaluation of CaCO_3_ Scale Inhibitors in Iron-Containing Brines

**DOI:** 10.1021/acsomega.6c08998

**Published:** 2026-10-01

**Authors:** Hugo N. S. Barros, Kaio A. B. Pereira, Matheus V. Oliveira, Aghata P. Felipe, Amaro L. G. O. A. Machado, Gustavo B. Silva, Juliana M. F. Façanha, Luiz C. M. Palermo, Claudia R. E. Mansur

**Affiliations:** † Programa de Engenharia Metalúrgica e de Materiais-PEMM/COPPE, 28125 Universidade Federal do Rio de Janeiro, Rio de Janeiro 21941-598, Brazil; ‡ Instituto de Macromoléculas/Laboratório de Macromoléculas e Coloides na Indústria de Petróleo, Universidade Federal do Rio de Janeiro, Rio de Janeiro 21941-598, Brazil; § Low Carbon & Subsurface Technology, Shell Brasil Petroleo LTDA, Rio de Janeiro 20031-170, Brazil

## Abstract

Scale inhibitors are widely used to control inorganic deposition in oil and gas production, but their laboratory evaluation is often performed under aerated conditions that may not adequately represent iron-containing brines. In this study, a modified Tube Block Test methodology was developed for dynamic scale-inhibition testing under controlled dissolved-oxygen conditions. Brines were prepared and transferred under a controlled atmosphere, and dissolved O_2_ concentrations ranging from 8500 to <10 μg/L were evaluated at 65 and 120 °C in tests lasting up to 6 h. A commercially available phosphonate-based inhibitor was used as an industry-qualified benchmark. The results indicated that dissolved oxygen strongly affected precipitation behavior and apparent inhibitor performance. At 65 °C under aerated conditions, the presence of iron substantially accelerated capillary blockage, whereas negligible differential-pressure variation was observed in the corresponding iron-free control. At 120 °C, rapid blockage also occurred in the iron-free control, consistent with an increased contribution from CaCO_3_ deposition. Reducing dissolved O_2_ to <10 μg/L delayed blockage in iron-containing brines and improved the apparent performance of the commercial inhibitor. Under oxygen-controlled conditions, the inhibitor at 250 mg/L maintained the differential-pressure increase below 1 psi throughout the 6 h test. These findings show that dissolved-oxygen control is essential for distinguishing iron-related interference from carbonate-scaling behavior during dynamic inhibitor qualification. The proposed methodology provides a reproducible approach for extended-duration evaluation of scale inhibitors in iron-containing brines under field-relevant operating conditions.

## Graphical abstract

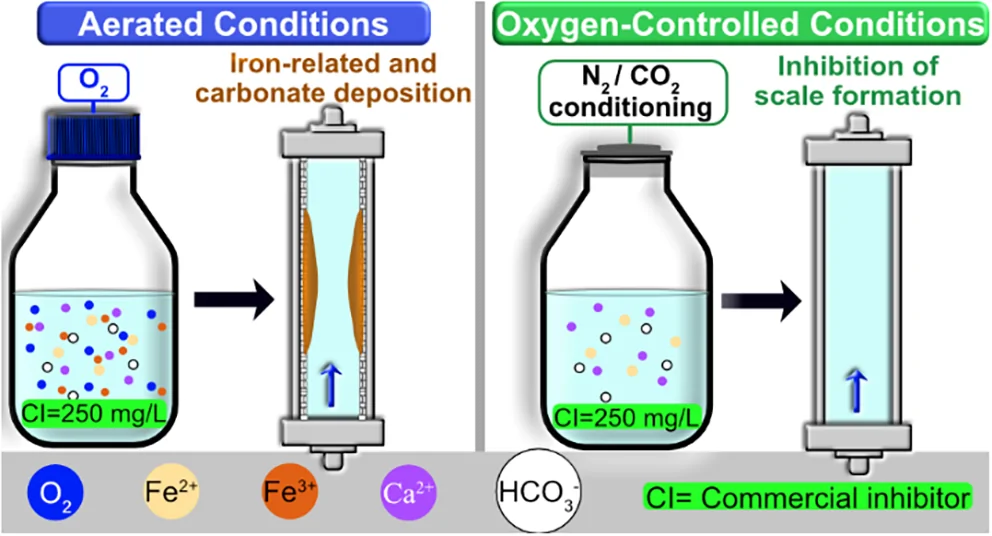

## Introduction

Inorganic scale deposition is a major flow-assurance challenge in oil and gas production because it restricts fluid flow, damages equipment, and generates substantial monitoring and remediation costs.^1,2^ Calcium carbonate is particularly relevant because many formation waters contain Ca^2+^ and HCO_3_^–^.^3,4^ During production, pressure and temperature changes promote CO_2_ degassing and shift carbonate equilibria toward CO_3_^2–^, increasing CaCO_3_ supersaturation and deposition.

Scale inhibitors such as polyaspartic acid (PASP), diethylenetriamine penta­(methylene phosphonic acid) (DTPMP), and diethylenetriamine penta­(methylene phosphonic acid)-based derivatives have been widely investigated for CaCO_3_ control and may achieve high inhibition efficiency at relatively low concentrations.^5–7^ However, their performance may decrease in the presence of dissolved or precipitated iron species.^7^ Although offshore reservoirs do not generally contain high iron concentrations, corrosion of pipelines and production equipment may release Fe^2+^ into produced fluids.^8^ Under reducing conditions, Fe^2+^ can remain dissolved or form sparingly soluble ferrous carbonate, whereas exposure to dissolved oxygen promotes its oxidation to Fe^3+^ and the subsequent formation of poorly soluble iron hydroxides and oxides.^8,9^ Under anoxic conditions, Fe^2+^ may also form poorly soluble ferrous hydroxide (Fe­(OH)_2_). Ferrous hydroxide may subsequently undergo the Schikorr reaction, forming magnetite (Fe_3_O_4_) and H_2_ and thereby providing an additional pathway for iron-containing solid formation.^10,11^ These solids may adhere to equipment surfaces, promote heterogeneous nucleation, or interact with scale inhibitors.

Iron is frequently omitted from synthetic brines used in laboratory qualification tests, particularly when present at relatively low concentrations. Nevertheless, even low iron concentrations may affect inhibitor evaluation because oxygen-induced changes in iron speciation modify precipitation pathways.^12^ As a result, tests conducted under conventional atmospheric conditions may produce misleading conclusions regarding inhibitor efficiency.^13^ This effect is particularly relevant for phosphonate-based formulations because iron species may interact with their functional groups or compete with mineral surfaces for inhibitor adsorption.

Dynamic flow-based methods, such as the Tube Block Test (TBT), are widely used to evaluate scale inhibitors under representative hydrodynamic, thermal, and pressure conditions. Standardized procedures, including NACE TM31105–2005,^14^ are commonly applied to assess carbonate and sulfate scale inhibition and determine minimum inhibitory concentrations under simulated field conditions. However, conventional configurations may not maintain controlled O_2_ levels and dissolved CO_2_ throughout brine preparation, transfer, and prolonged testing. This limitation becomes critical in iron-containing systems because changes in iron speciation may be incorrectly interpreted as scale inhibitor failure.^15,16^

Previous studies under high-temperature and high-pressure conditions have demonstrated that maintaining iron predominantly in the ferrous state requires oxygen-controlled environments.^12,17^ Oxygen-free nitrogen sparging, sealed vessels, and continuous nitrogen blankets have been used to limit oxygen ingress. However, most reported configurations provide limited information on simultaneous O_2_ control, CO_2_ equilibration, closed brine transfer, and extended-duration injection. Table 1 compares these approaches with the methodology proposed in this study.

**Table 1:** Summary of Laboratory Test Conditions and Oxygen Removal Methods for Evaluating Scale Inhibitor Performance in the Presence of Dissolved Iron

Authors	Test type	Brine storage	Method for removing O_2_	O_2_ level	Maximum time (min)	Iron concentration (mg/L)
Graham et al. (2003)^15^	Dynamic flow loop	Screw cap bottles with O_2_ trap and constant N_2_ sparging	Sparging the brine with OFN.	<5 μg/L	∼100	Up to 10
	*P* = 1500 psi, *T* = 98 °C					
Guan et al. (2009)^8^	Modified corrosion-kettlecell	1 L culture vessel with reflux condenser	Sparging with CO_2_ followed by N_2_	Not reported (significantly limited ingress)	∼172	150
	*P* = Atmospheric, *T* = 50 °C					
Daniels and Feasey (2020)^18^	Dynamic flow loop	Screw cap bottle with N_2_ “blanket”	Brine sparged with N_2_	Not reported	∼ 100	250
	*P* = 50 psi, *T* = 98 °C					
This work	Dynamic flow loop	Stainless steel 2 L piston accumulators	Brine sparged with N_2_	<10 μg/L	∼360	8.7
	*P* = 1073 psi, *T* = 65 and 120 °C					

In this study, a modified TBT methodology was developed to control dissolved oxygen while maintaining CO_2_ equilibrium during brine preparation, transfer, and dynamic testing. The method was designed to minimize Fe^2+^ oxidation and reduce interference from iron-related precipitation in the evaluation of CaCO_3_ scale inhibitors. Experiments were conducted using iron-containing brines under representative reservoir and surface-processing conditions, with an industry-qualified phosphonate-based inhibitor used as the benchmark.

## Experimental Section

### Materials

All chemicals were used as received without further purification. A commercial scale inhibitor (CI), supplied by an industry partner, was used as an industry-qualified benchmark. According to the supplier, the commercial formulation contains phosphonate and/or phosphino-polycarboxylate chemistries combined with auxiliary components intended to improve scale inhibition, tolerance to iron species, and operational performance in complex brines. The exact composition of the formulation was proprietary. All inhibitor concentrations reported in this study refer to the commercial formulation as received rather than to the concentration of active ingredient. ACS reagent-grade sodium chloride (NaCl), potassium chloride (KCl), barium chloride dihydrate (BaCl_2_·2H_2_O), magnesium chloride hexahydrate (MgCl_2_·6H_2_O), strontium chloride hexahydrate (SrCl_2_·6H_2_O), calcium chloride dihydrate (CaCl_2_·2H_2_O), sodium sulfate (Na_2_SO_4_), sodium bicarbonate (NaHCO_3_), sodium acetate (CH_3_COONa), and ferrous chloride tetrahydrate (FeCl_2_·4H_2_O) were obtained from Sigma-Aldrich. Concentrated acetic acid (98%) was supplied by Merck, whereas sodium hydroxide (NaOH) and hydrochloric acid (HCl, 37%) were purchased from Synth. Commercial nitrogen (N_2_) and carbon dioxide (CO_2_), used for brine purging and atmospheric control, were supplied by Air Liquide.

### Brine Preparation

Two synthetic brines representative of a selected field were prepared using deionized water, corresponding to formation brine and injection brine, according to the composition presented in Table 2. The formation brine was formulated to contain only the target cations, with chloride as the sole anion. In contrast, the injection brine was prepared to contain only the anions relevant to the scaling process, with sodium as the sole cation, while other multivalent cations were excluded. The molar masses of the hydrated salts, including their crystallization waters, were used to calculate the ionic concentrations.

**Table 2:** Brine Composition

Formation Brine		Injection Brine		Unit
Cl^–^	93,061.0	Cl^–^	75,216.0	mg/L
Na^+^	48,778.7	Na^+^	49,167.3	
Ca^2+^	6,070.0	SO_4_^2–^	61.0	
Mg^2+^	1,934.0	HCO_3_^–^	942.0	
Sr^2+^	710.0	CH_3_COO^–^	11.3	
K^+^	812.0			
Ba^2+^	256.0			
Fe^2+^	17.4			

### Preparation of Brine under Atmospheric Conditions

All salts were dissolved during brine preparation, except for ferrous chloride tetrahydrate (FeCl_2_·4H_2_O), which was added to the formation brine immediately before injection into the TBT system to minimize Fe^2+^ oxidation. The Fe^2+^ concentration in the formation brine was 17.4 mg/L. Because the formation and injection brines were mixed at equal flow rates (1:1), the nominal iron concentration entering the precipitation section of the TBT was 8.7 mg/L. Iron speciation was not directly measured during the dynamic experiments. The pH of the injection brine was adjusted to 6.5 by controlled CO_2_ sparging to establish the target carbonate–bicarbonate equilibrium. The pH of the formation brine was not adjusted; after the addition of FeCl_2_·4H_2_O, its pH remained above 3.0. Table 3 summarizes the experimental conditions, including the CO_2_ concentration, injection-brine pH, operating temperature, and pressure. Tests were conducted at 65 and 120 °C to represent reservoir and high-temperature surface-processing conditions, respectively, while the system pressure was maintained at 1073 psi.

**Table 3:** Experimental Conditions Used in the Tube Block Test

Experimental Conditions		Unit
CO_2_	10	%
pH	6.5	-
Temperature	65 and 120	°C
Pressure	1073	psi

### Preparation of Brines under Oxygen-Controlled Conditions

For oxygen-controlled experiments, the brines were conditioned inside a glovebox purged with N_2_. The solutions were sparged with N_2_ until the dissolved O_2_ concentration reached either approximately 2000 μg/L or below 10 μg/L, depending on the experimental condition, as monitored using an M400 Type 2 transmitter equipped with an InPro 6860*i*/12/120 sensor (Mettler-Toledo). The pH of the injection brine was then adjusted to 6.5 by controlled CO_2_ sparging, monitored using an M400 Type 3 transmitter and an InPro 5000*i*/12/120 CO_2_ sensor. FeCl_2_·4H_2_O was added to the formation brine immediately before transfer to minimize Fe^2+^ oxidation. The conditioned brines were transferred by vacuum-assisted filling into stainless-steel displacement cylinders, which were sealed and connected to the TBT system without exposure to the external atmosphere. Figure 1 illustrates the conditioning and transfer procedure.

**Figure 1:**
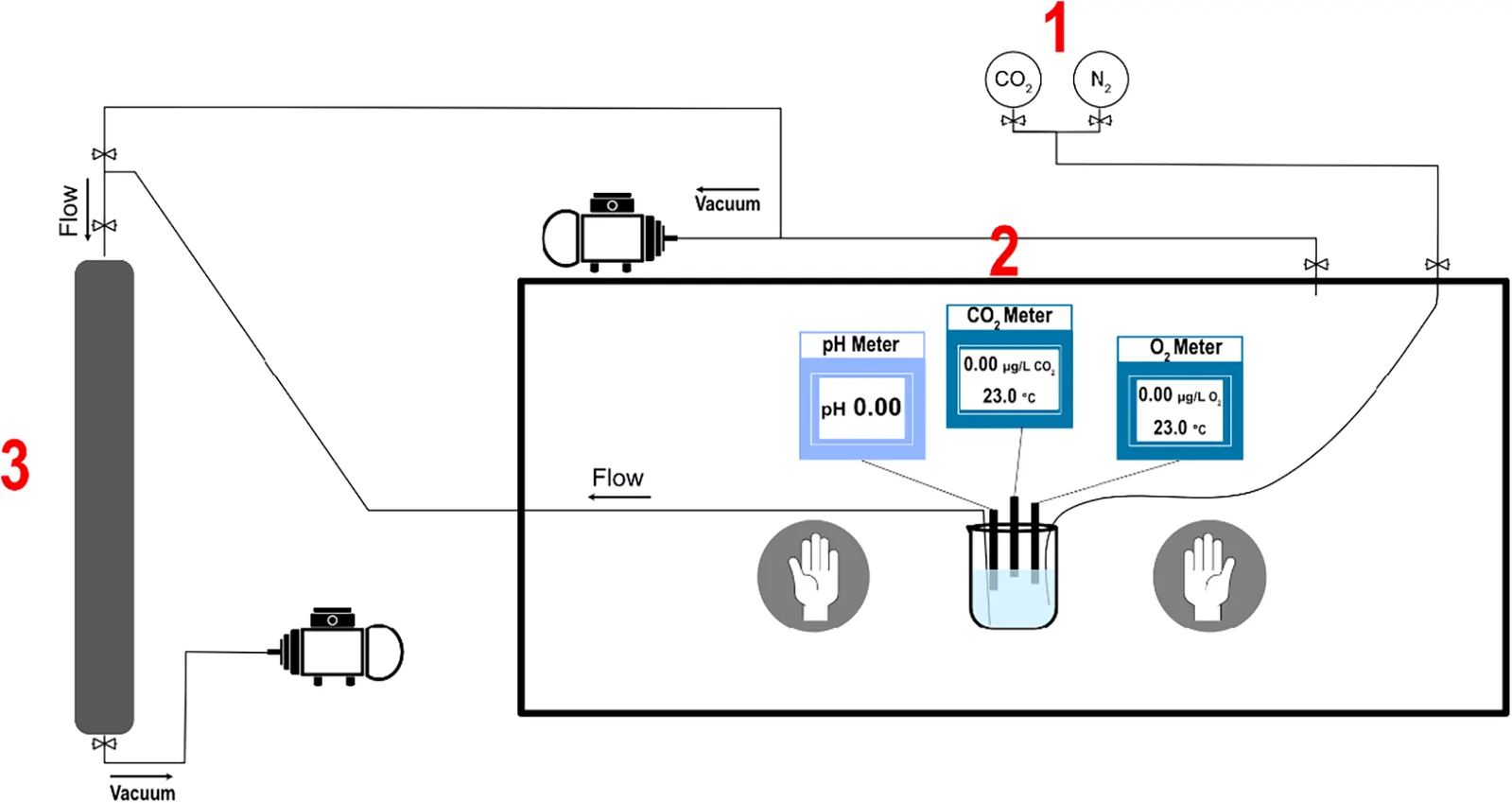
Schematic representation of the glovebox system used for brine conditioning and transfer under oxygen-controlled conditions. Note: Glovebox: (1) N_2_ and CO_2_ gas outlets; (2) pH, CO_2_, and O_2_ sensors; (3) fluid trapping cylinder.

### Dynamic Scale Inhibition Test (Tube Block Test)

Dynamic scale inhibition experiments were performed using a TBT apparatus based on NACE TM31105–2005 standard,^14^ modified for dissolved CO_2_ retention and operation under controlled low-oxygen conditions (Figure 2).

**Figure 2:**
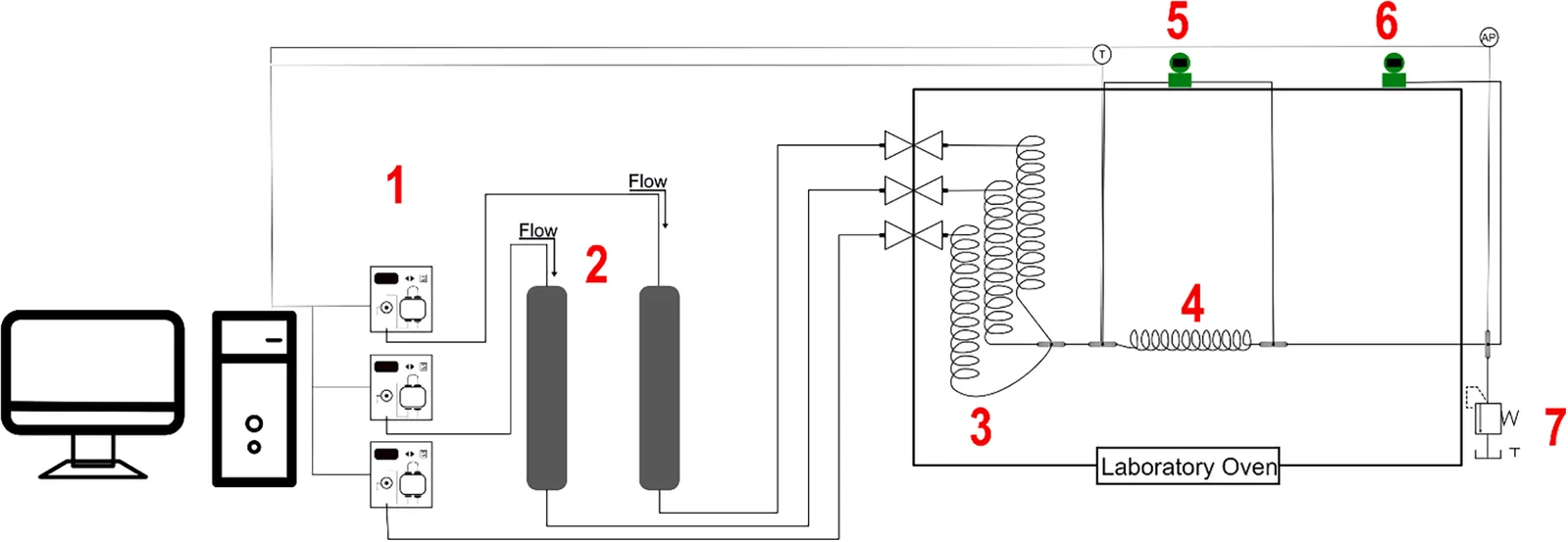
Schematic representation of the Tube Block Test apparatus used for dynamic scale-inhibition experiments. Note: TBT: (1) chromatographic pumps; (2) stainless-steel displacement cylinders with internal pistons; (3) heating coils (6 m length); (4) precipitation capillary (1 m length and 0.59 mm inside diameter); (5) differential pressure transducer; (6) absolute pressure transducer; (7) back-pressure relief valve.

All flow lines, including the heating coils and precipitation capillary, were installed inside a temperature-controlled oven to ensure thermal stability during the experiments. Pressure and flow data were continuously monitored and recorded using dedicated acquisition software. During the tests, the formation and injection brines were each pumped at 5 mL/min, resulting in a total flow rate of 10 mL/min. The baseline differential pressure was established after 1.5 min of flow, once the signal had stabilized. Control experiments were terminated when the differential pressure increased by 5 psi above the baseline, whereas inhibitor-containing experiments were terminated at a 1 psi increase. Test duration varied according to the experimental objective. The qualification criterion adopted in this study required the inhibitor to delay capillary blockage to at least three times the control blocking time while maintaining the differential-pressure increase below 1 psi relative to the baseline. All experiments were performed in triplicate, and the relative standard deviation of the blocking time was below 5%. LOESS smoothing was applied to all graphs to reduce local fluctuations and improve visualization of the underlying trends. Representative differential-pressure profiles are shown. Blocking times are reported as the mean ± standard deviation of three independent experiments. Temperatures and inhibitor concentrations were selected based on representative field conditions, preliminary screening, and the qualification protocol adopted for the project. The dissolved oxygen concentration was measured immediately before transfer to the TBT system and was not continuously monitored during the dynamic experiments. After filling, the stainless-steel displacement cylinders were sealed and maintained under positive pressure to minimize atmospheric gas ingress. Therefore, the reported dissolved-O_2_ concentrations represent the initial experimental conditions.

## Results and Discussion

In conventional dynamic studies on sulfate and carbonate scaling, dissolved oxygen is rarely treated as a critical variable because the principal scaling ions, such as Ca^2+^, Ba^2+^, and Sr^2+^, do not participate in redox reactions under typical reservoir conditions. However, in iron-containing brines, this assumption no longer holds, as dissolved oxygen becomes a dominant factor governing precipitation behavior. Ferrous iron is rapidly oxidized to ferric iron even under ambient oxygen exposure and Fe^3+^ readily undergoes hydrolysis and precipitation as ferric hydroxides, irrespective of brine compatibility tests. These species may also act as coprecipitation agents or nucleation sites for other ions.^7,19^

From a methodological standpoint, controlling the gas composition of the system is therefore essential to delay Fe^2+^/Fe^3+^ redox transformations, stabilize iron speciation, and preserve the carbonate-bicarbonate equilibrium with CaCO_3_ formation, one of the most common scales in oilfield production systems. To isolate and quantify the effects of iron ions and dissolved oxygen on dynamic precipitation behavior, a series of Tube Block Test experiments was conducted using bicarbonate-rich brines at a fixed pressure of 1073 psi and at two representative temperatures: 65 and 120 °C, corresponding to reservoir and separator conditions for heavier oils, respectively. The dissolved oxygen concentration varied from ambient conditions, 8500 μg/L (ambient pressure without further sparging of N_2_ on the brine) and with the preparation in the glovebox, <10 μg/L.

### Effect of Iron and Oxygen at 65 °C

The influence of iron and dissolved oxygen was evident in the dynamic experiments conducted at 65 °C. Under aerated conditions, the iron-containing control exhibited a pronounced increase in differential pressure (dP), leading to capillary blockage. As shown in Figure 3, the differential pressure reached values above 12 psi after approximately 120 min. In contrast, the corresponding control experiment performed without (w/o) iron showed negligible changes in dP throughout the test, suggesting that no significant CaCO_3_ precipitation occurred under these conditions. The observed behavior suggests a predominant contribution from iron-related precipitation rather than CaCO_3_ deposition under the evaluated conditions.

**Figure 3:**
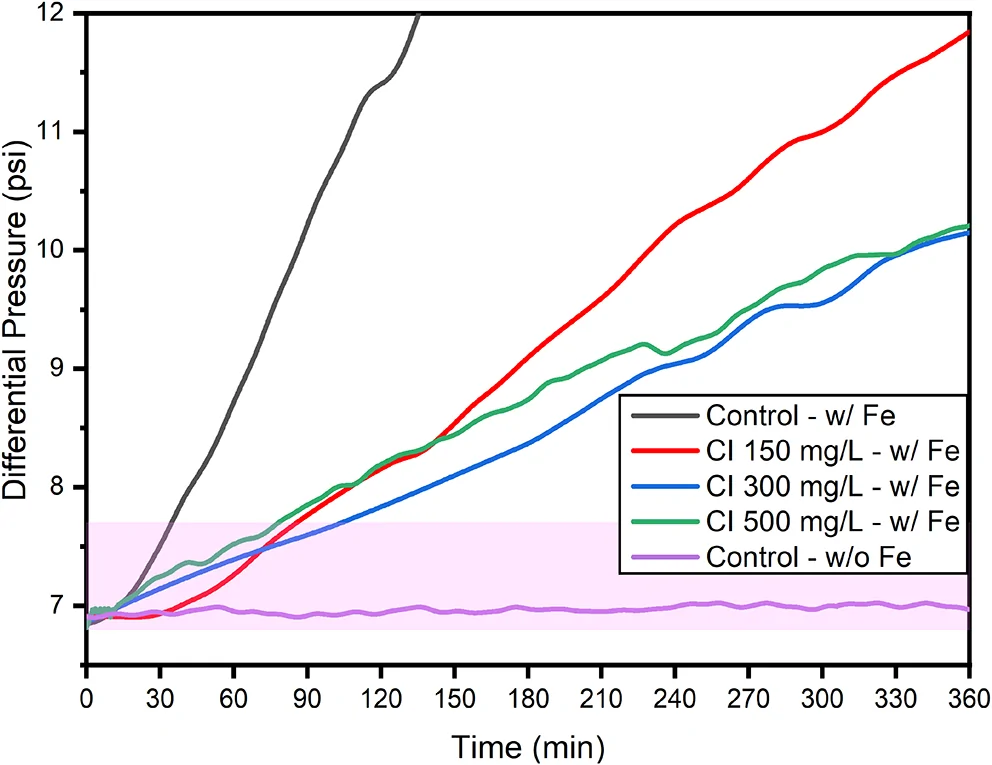
Dynamic test results for the control experiments (with and without iron) and for the commercial inhibitor (CI) at 65 °C under atmospheric conditions. Note: The shaded region represents the acceptance range corresponding to a differential-pressure increase of less than 1 psi above the initial baseline.

Consequently, under aerated conditions at 65 °C, the response of the commercial inhibitor appears to reflect its interaction with iron-related precipitation processes rather than exclusively its ability to control CaCO_3_ scaling.^20^ This interpretation is also supported by the distinct differential-pressure profiles associated with these precipitation processes. Carbonate precipitation typically produces a relatively sharp, exponential-like increase in differential pressure,^18^ consistent with its rapid precipitation kinetics,^21^ whereas deposition processes involving iron species generally exhibit a more gradual increase in differential pressure, as previously reported in the literature.^13,16^

The differential-pressure profiles obtained at the three inhibitor concentrations (150, 300, and 500 mg/L) were very similar. Although an improvement in performance was observed when the inhibitor concentration was increased from 150 to 300 mg/L, a further increase to 500 mg/L resulted in a slight decrease in efficiency. This behavior may be associated with aggregation or flocculation of components of the commercial inhibitor at higher concentrations, although this mechanism was not directly investigated in the present study. Overall, the results suggest that the optimal inhibitor concentration under the evaluated conditions is close to 300 mg/L. Although the inhibitor partially reduced the rate of differential-pressure increase, it did not completely prevent capillary blockage under aerated conditions. Such behavior indicates a limitation of the inhibitor in controlling precipitation processes associated with oxidized iron species. Previous studies have similarly reported strong adsorption of phosphonate-based inhibitors onto the surfaces of metal hydroxides, including ferric hydroxide particles.^12,22^ Although subsurface conditions are typically anoxic, the observed behavior suggests that the commercial inhibitor may be adversely affected by iron oxides, potentially favoring the formation of iron-bearing solids throughout the production system.

Overall, the results obtained at 65 °C may indicate that iron oxidation products contributed more strongly to capillary blockage than CaCO_3_ precipitation under the evaluated conditions. The negligible differential-pressure increase observed in the iron-free control indicates that CaCO_3_ deposition was not sufficient to cause measurable blockage during the test period. Therefore, under these specific conditions, iron-control strategies involving stabilizing or sequestering agents, including citrate,^23^ HEDP (1-hydroxyethylidene-1,1-diphosphonic acid),^24^ HEDTA (*N*-(2-hydroxyethyl)­ethylenediamine-*N*,*N*′,*N*′-triacetic acid), and EDTA (ethylenediaminetetraacetic acid),^25^ may be more relevant than increasing the dosage of a conventional CaCO_3_ scale inhibitor.

### Effect of Iron and Oxygen at 120 °C

At 120 °C, the precipitation behavior changed markedly, consistent with the increased CaCO_3_ scaling tendency at elevated temperature.^26^ As shown in Figure 4, the rapid blockage observed in the iron-free control is consistent with an increased contribution from CaCO_3_ deposition at elevated temperature. A commercial inhibitor concentration of 250 mg/L was selected as the benchmark concentration for the subsequent experiments.

**Figure 4:**
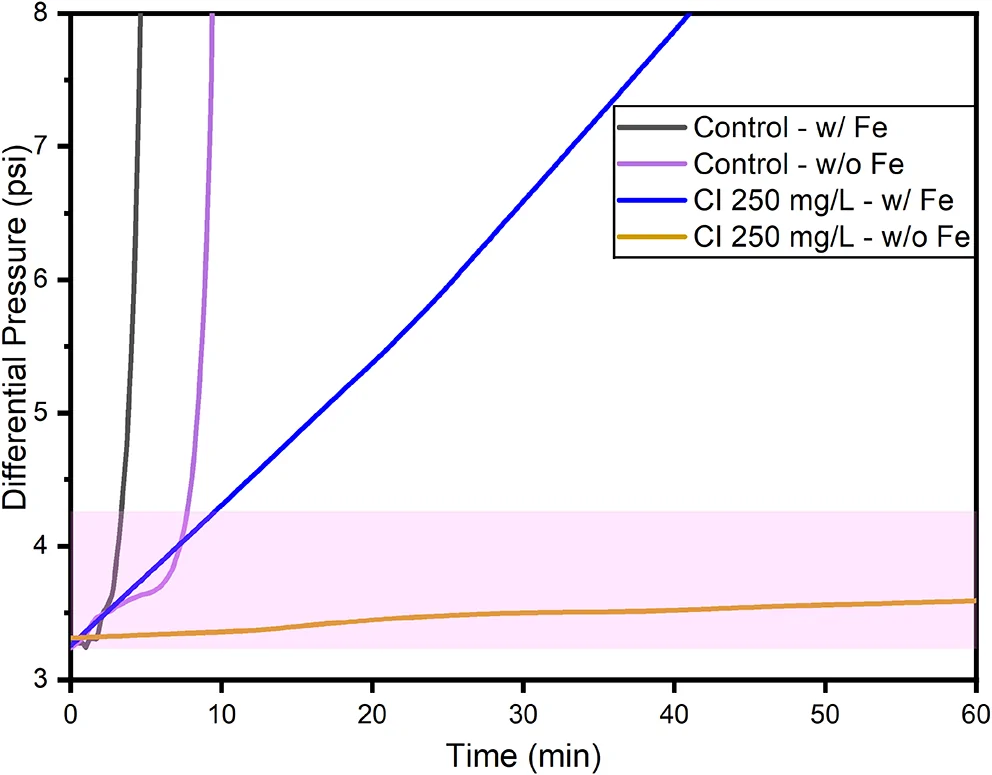
Dynamic tube block test results for control experiments (with and without iron) and for the commercial inhibitor (250 mg/L) at 120 °C under atmospheric conditions. Note: The shaded region represents the acceptance range corresponding to a differential-pressure increase of less than 1 psi above the initial baseline.

In the presence of iron, capillary blockage occurred more rapidly, suggesting that iron-containing species accelerated the overall deposition process. This behavior indicates that carbonate scaling and iron-related precipitation may occur simultaneously at elevated temperatures, highlighting the need to distinguish their respective contributions when interpreting dynamic tests with commercial inhibitors.

In iron-free conditions, no appreciable increase in differential pressure was observed, indicating that the commercial inhibitor effectively controlled deposition under the evaluated conditions. In contrast, the presence of iron resulted in a progressive increase in differential pressure, suggesting reduced inhibitor performance. This behavior is consistent with the “poisoning effect” reported for phosphonate-based inhibitors, in which interactions between iron species and phosphonate functional groups decrease the amount of inhibitor available for adsorption onto active crystal-growth sites. Similar effects have been reported for phosphonate inhibitors evaluated in iron-containing brines.^15,17,19^

Therefore, the results suggest that the phosphonate-based commercial inhibitor effectively controlled carbonate-related deposition under the evaluated high-temperature conditions. In the presence of iron and dissolved oxygen, however, its performance decreased, consistent with interactions involving iron oxidation products. Under these conditions, complementary iron-control additives may help stabilize or sequester dissolved iron species, thereby maintaining iron in solution and preserving inhibitor performance. Because the deposited solids were not directly characterized, the proposed phase assignments should be regarded as mechanistic interpretations based on the differential-pressure profiles, brine composition, and comparison between iron-containing and iron-free controls.

### Effect of Dissolved Oxygen Concentration

To assess how dissolved oxygen affects inhibitor performance, dynamic experiments were conducted at 120 °C using iron-containing brines with dissolved O_2_ concentrations of 8500, 2000, and <10 μg/L. The results are shown in Figure 5.

**Figure 5:**
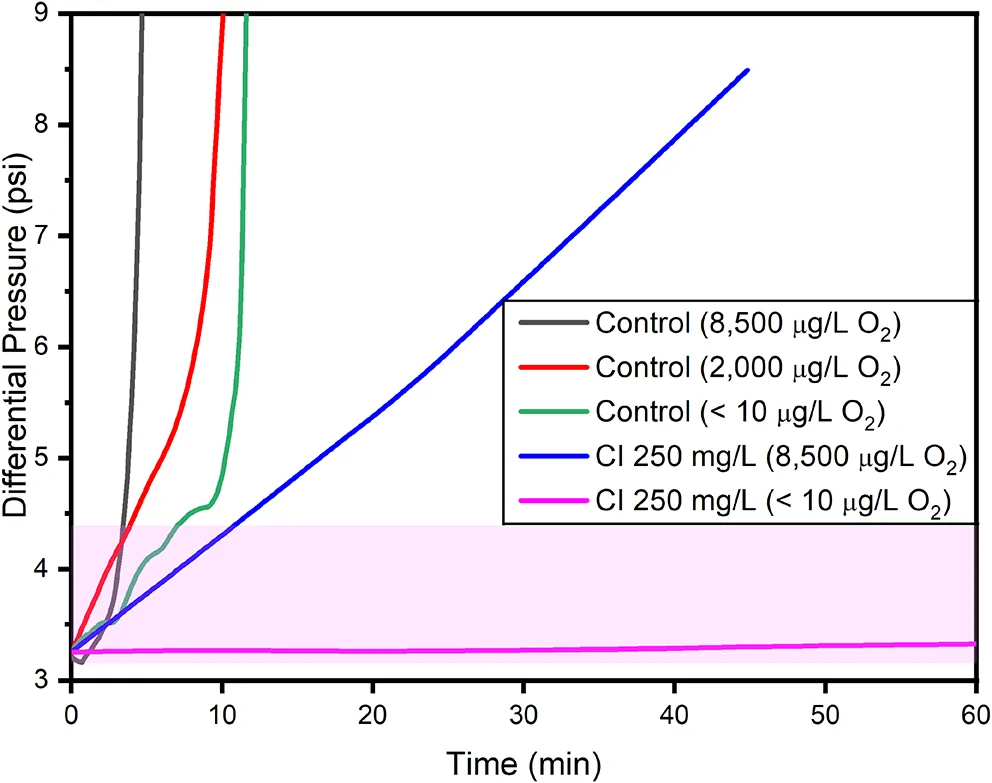
Dynamic results for control experiments and the commercial inhibitor under different dissolved oxygen concentrations at 120 °C. Note: The shaded region represents the acceptance range corresponding to a differential-pressure increase of less than 1 psi above the initial baseline.

Under high oxygen concentration, representative of preparing brine in atmospheric conditions without further treatment (8500 μg/L), the control experiment with iron resulted in the fastest capillary blockage. Reducing oxygen concentration (2000 μg/L) resulted in a delay in the onset of capillary blockage. When the oxygen concentration was reduced to below 10 μg/L, a pronounced delay in precipitation was observed, indicating that dissolved oxygen plays a key role in accelerating precipitation and capillary blockage.

This behavior is consistent with the formation of poorly soluble iron species, including iron hydroxides and oxides, under oxidizing conditions. These solids may act as heterogeneous nucleation sites and promote deposit growth within the system. The commercial inhibitor (250 mg/L) exhibited markedly different behavior at high (8500 μg/L) and ultralow (<10 μg/L) dissolved O_2_ concentrations. At 8500 μg/L O_2_, the differential pressure increased continuously, indicating reduced inhibitor performance. In contrast, at <10 μg/L O_2_, the differential pressure remained nearly constant throughout the experiment and satisfied the dynamic validation criterion. Similar behavior has been reported in previous studies, in which dissolved oxygen promoted iron oxidation and reduced the performance of phosphonate-based scale inhibitors.^8,16^ Although the Schikorr reaction may occur under anoxic conditions, no progressive increase in differential pressure was observed in the inhibitor-containing experiment at <10 μg/L O_2_. Under the CO_2_-rich conditions and the temperature and pressure employed in this study, iron speciation may have favored the formation of ferrous carbonate (FeCO_3_) rather than magnetite (Fe_3_O_4_).^25^ The stable differential-pressure profile further suggests that the commercial inhibitor effectively suppressed the deposition of carbonate-containing solids under these conditions.^27^ These findings reinforce the importance of oxygen control when evaluating inhibitors in iron-containing systems.

### Extended-Duration Validation

Evaluating inhibitor performance over extended periods is important to ensure effectiveness throughout the residence time of fluids in flowlines, risers, and processing facilities.^28^ Depending on tieback distance, residence times may reach 8 h. A common qualification criterion requires the inhibitor to maintain the differential-pressure increase below the specified limit for at least three times the control blocking time. In this study, the tests were extended to 6 h to reproduce field operating conditions provided by an oil and gas operator. Under the adopted qualification criterion, the product was considered acceptable when the differential-pressure increase remained below 1 psi relative to the baseline throughout the test.

As shown previously, rapid capillary blockage occurred in the absence of inhibitor even under oxygen-depleted conditions, consistent with the increased CaCO_3_ scaling tendency at 120 °C. As shown in Figure 6, the commercial inhibitor at 250 mg/L maintained the differential pressure nearly constant throughout the 6 h test. This result demonstrates the importance of extended-duration testing for reproducing field-relevant residence times and assessing inhibitor performance beyond short-term screening criteria.

**Figure 6:**
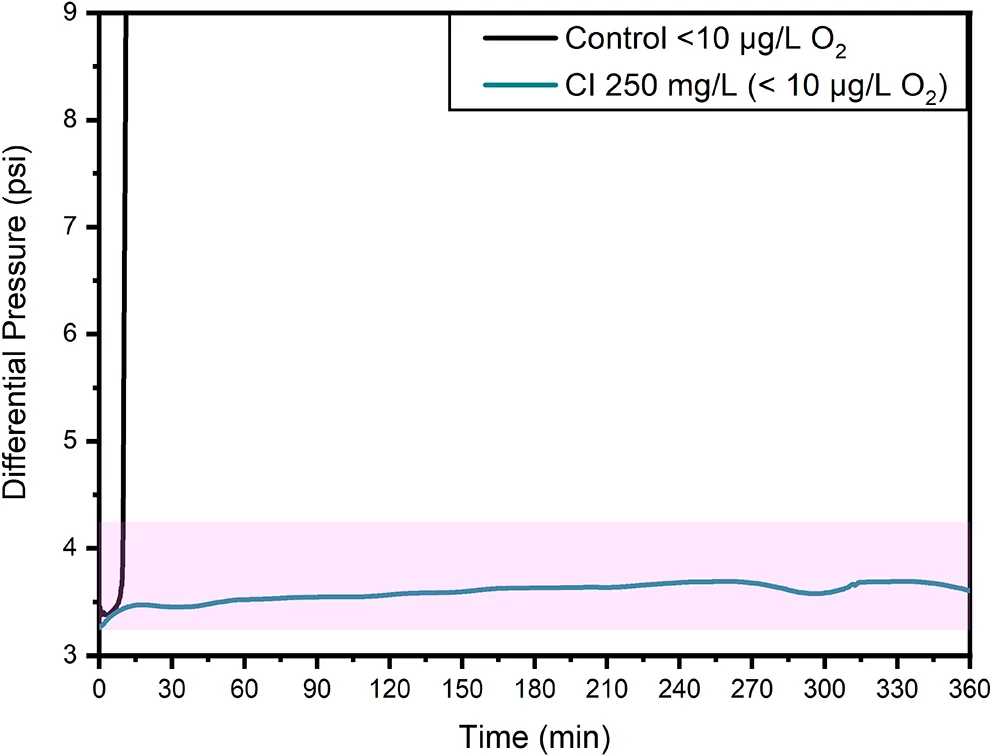
Dynamic test results for the control experiment and CI at 250 mg/L under low-oxygen conditions (<10 μg/L O_2_) at 120 °C during a 6 h test. Note: The shaded region represents the acceptance range corresponding to a differential-pressure increase of less than 1 psi above the initial baseline.

### Methodological Implications

Taken together, these results indicate that dissolved oxygen is a critical and often overlooked variable in dynamic scale-inhibition testing of iron-containing brines. Conventional aerated tests may confound precipitation associated with iron oxidation with reduced scale-inhibitor performance, potentially leading to inaccurate conclusions regarding inhibitor efficiency and dosage requirements. By incorporating strict oxygen control, the proposed methodology reduces interference from iron-related precipitation and enables a more reliable and reproducible evaluation of scale inhibitors in iron-containing systems.

## Conclusions

This study showed that dissolved oxygen is a critical variable in dynamic scale-inhibition testing of iron-containing brines, affecting precipitation behavior and the apparent performance of the commercial inhibitor evaluated in this study. Under aerated conditions, the observed blockage behavior was consistent with the formation of poorly soluble iron-containing species, which may have contributed to accelerated capillary blockage and reduced the apparent performance of the commercial inhibitor, potentially leading to inaccurate laboratory assessments under the evaluated conditions.

Maintaining the dissolved O_2_ concentration below 10 μg/L while preserving CO_2_ equilibrium reduced the apparent interference associated with iron-related precipitation and enabled a more representative evaluation of the tested inhibitor formulation. At 65 °C, the blockage behavior was primarily consistent with iron-related deposition, whereas at 120 °C, the results indicated a greater contribution from carbonate scaling. Under oxygen-controlled conditions, the commercial inhibitor at 250 mg/L maintained the differential-pressure increase below 1 psi relative to the baseline throughout the 6 h test.

These findings highlight the importance of dissolved oxygen control when evaluating scale inhibitors in iron-containing brines under the conditions investigated in this study. The proposed methodology provides a reproducible approach for extended-duration dynamic testing and may improve the interpretation of inhibitor performance under field-relevant operating conditions. However, because only one commercial inhibitor formulation was evaluated, further studies involving inhibitors with different chemistries are required to determine the broader applicability of these findings.

## References

[1] Husna U. Z., Elraies K. A., Shuhili J. A. B. M., Elryes A. A. (2022). A review: the utilization
potency of biopolymer as an eco-friendly scale inhibitors. J. Pet. Explor. Prod. Technol..

[2] Baraka-Lokmane S., Sorbie K., Poisson N., Kohler N. (2009). Can. Green
Scale Inhibitors
Replace Phosphonate Scale Inhibitors?: Carbonate Coreflooding Experiments. Pet. Sci. Technol..

[3] MacAdam, J. ; Jarvis, P. Water-Formed Scales and Deposits. In Miner. Scales Deposits; Elsevier, 2015; pp 3–23 10.1016/B978-0-444-63228-9.00001-2.

[4] Li H., Cai T., Yuan B., Li R., Yang H., Li A. (2015). Flocculation
of both kaolin and hematite suspensions using the starch-based flocculants
and their floc properties. Ind. Eng. Chem. Res..

[5] Chen Y., Chen X., Liang Y., Gao Y. (2021). Synthesis of polyaspartic
acid-oxidized starch copolymer and evaluation of its inhibition performance
and dispersion capacity. J. Dispersion Sci.
Technol..

[6] Kan A. T., Oddo J. E., Tomson M. B. (1994). Formation of Two Calcium Diethylenetriaminepentakis­(methylene
phosphonic acid) Precipitates and Their Physical Chemical Properties. Langmuir.

[7] Stoppelenburg, L. S. ; Yuan, M. In The Performance of Barlum Sulfate Inhibitors in Iron Containing Waters in Both Aerated and Anaerobic Systems; CORROSION 2000, AMPP: Orlando, Florida, 2000.

[8] Guan H., Cole G., Clark P. (2009). Inhibitor
Selection for Iron-Scale
Control in MEG Regeneration Process. SPE Prod.
Oper..

[9] Kan, A. T. ; Fu, G. ; Shen, D. ; Al-Saiari, H. ; Tomson, M. B. In Enhanced Inhibitor Treatments with the Addition of Transition Metal Ions; SPE International Oilfield Scale Conference SPE, 2008 10.2118/114060-MS.

[10] Ma M., Zhang Y., Guo Z., Gu N. (2013). Facile synthesis of
ultrathin magnetic iron oxide nanoplates by Schikorr reaction. Nanoscale Res. Lett..

[11] Kiran R., Saif M., Wood D. A., Gupta S. D., Kumar A., Upadhyay R., Rajak V. K. (2025). Geological Hydrogen Prospectivity
of Iron Ore Deposits in India Based on Semiquantitative Analysis. ACS Omega.

[12] Zhang Z., Zhang P., Li Z., Kan A. T., Tomson M. B. (2018). Laboratory
Evaluation and Mechanistic Understanding of the Impact of Ferric Species
on Oilfield Scale Inhibitor Performance. Energy
Fuels.

[13] Shen D., Shcolnik D., Perkins R., Taylor G., Brown M. (2012). Evaluation
of scale inhibitors in marcellus high-iron waters. Oil Gas Facil..

[14] NACE 31105–2005 . Dynamic Scale Inhibitor Evaluation Apparatus and Procedures in Oil and Gas Production. Texas: 2005.

[15] Graham, G. M. ; Stalker, R. ; McIntosh, R. In The Impact of Dissolved Iron on the Performance of Scale Inhibitors Under Carbonate Scaling Conditions; International Symposium on Oilfield Chemistry SPE, 2003 10.2118/80254-MS.

[16] Dyer, S. J. ; Graham, G. M. In Influence of Iron on Scale Inhibitor Performance and Carbonate Scale Formation; 11th NIF International Oil Field Chemical Symposium Heriot-Watt University: Fagernes, 2000; pp 180–189.

[17] Zhang P., Zhang Z., Liu Y., Kan A. T., Tomson M. B. (2019). Investigation
of the impact of ferrous species on the performance of common oilfield
scale inhibitors for mineral scale control. J. Pet Sci. Eng..

[18] Daniels, J. K. ; Feasey, N. In Development of Scale Inhibitor for High Salinity High Iron Containing Brines; International Oilfield Scale Conference and Exhibition SPE, 2020 10.2118/200655-MS.

[19] Zhang, Z. ; Liu, Y. ; Dai, Z. ; Bhandari, N. ; Zhang, F. ; Yan, F. In Impact of FeIII/FeII on Scale Inhibition; SPE International Oilfield Scale Conference and Exhibition SPE, 2016 10.2118/179905-MS.

[20] Lu, H. ; Alattal, F. ; Ruggeri, J. ; Brooks, J. ; Lynn, J. ; Hughes, B. In Iron Effects on Scale Inhibitor Performances for Laboratory and Field Operations; CORROSION 2016, AMPP, 2016.

[21] Probst J., Borca C. N., Newton M. A., van Bokhoven J., Huthwelker T., Stavrakis S., deMello A. (2021). In Situ X-ray Absorption
Spectroscopy and Droplet-Based Microfluidics: An Analysis of Calcium
Carbonate Precipitation. ACS Meas. Sci. Au.

[22] Nowack B., Stone A. T. (1999). The Influence of
Metal Ions on the Adsorption of Phosphonates
onto Goethite. Environ. Sci. Technol..

[23] Shen D., Shcolnik D., Perkins R., Taylor G., Brown M. (2012). Evaluation
of Scale Inhibitors in Marcellus High-Iron Waters. Oil Gas Facil..

[24] Hamouda A. A. (1990). Insight
Into Sulfate in High-Salinity Producers and Selection of Scale Inhibitor. SPE Prod. Eng..

[25] Kamal M. S., Hussein I., Mahmoud M., Sultan A. S., Saad M. A. S. (2018). Oilfield
scale formation and chemical removal: A review. J. Pet Sci. Eng..

[26] Mazumder M. A. J. (2020). A review
of green scale inhibitors: Process, types, mechanism and properties. Coatings.

[27] Matamoros-Veloza A., Stawski T. M., Vargas S., Neville A. (2023). Study of a Local Structure
at the Interface between Corrosion Films and Carbon Steel Surface
in Undersaturated CO_2_ Environments. ACS Omega.

[28] Kelland, M. A. Production Chemicals for the Oil and Gas Industry, 2nd ed.; Taylor & Francis Group: New York, 2014.

